# Vaccination status and self-reported side effects after SARS-CoV-2 vaccination in relation to psychological and clinical variables in patients with multiple sclerosis

**DOI:** 10.1038/s41598-024-62541-x

**Published:** 2024-05-28

**Authors:** Katja Burian, Felicita Heidler, Niklas Frahm, Michael Hecker, Silvan Elias Langhorst, Pegah Mashhadiakbar, Barbara Streckenbach, Julia Baldt, Janina Meißner, Jörg Richter, Uwe Klaus Zettl

**Affiliations:** 1 Neuroimmunology Section, Department of Neurology, Rostock University Medical Center, Gehlsheimer Straße 20, 18147 Rostock, Germany; 2 Department of Neurology, Ecumenic Hainich Hospital gGmbH, Pfafferode 102, 99974 Mühlhausen, Germany; 3 Faculty of Health Sciences, University of Hull, Cottingham Rd, Hull, HU6 7RX UK; 4 Durham Law School, The Palatine Centre, Durham University, Stockton Rd, Durham, DH1 3LE UK

**Keywords:** SARS-CoV-2, Multiple sclerosis, Vaccination willingness, Vaccination status, Vaccination hesitancy, Side effects, Multiple sclerosis, Vaccines

## Abstract

The recent SARS-CoV-2 pandemic and the vaccination campaign posed a challenge to patients with autoimmune disease, such as multiple sclerosis (MS). We aimed for investigating whether psychological/sociodemographic/clinical characteristics of MS patients are associated with SARS-CoV-2 vaccination status and self-reported vaccination side effects (SEs). We have asked patients with MS about their willingness to receive recommended standard vaccinations pre-pandemically since June 2019. Between 10/2021 and 01/2022, we surveyed 193 of these MS patients about their current SARS-CoV-2 vaccination status, their perception of vaccination-related SEs, and reasons for and against SARS-CoV-2 vaccination. 75.6% of the patients declared their willingness to receive standard vaccinations before the pandemic. 84.5%, 78.2%, and 13.0% of the patients had received the first, second, and third SARS-CoV-2 vaccination, respectively, until the follow-up survey. The most common reason for not getting vaccinated against SARS-CoV-2 was concern about possible side effects (82.1%), followed by the belief that the vaccines had not been adequately tested (64.3%). Vaccination-related SEs were reported by 52.8% of the patients. Younger age, higher education, lower degree of disability, relapsing disease course, shorter disease duration, not receiving a disease-modifying therapy and higher anxiety and depression levels were associated with the occurrence of certain vaccination-related SEs. Concerns about novel vaccines are widespread among MS patients and necessitate targeted education of the patients, especially to those with more severe psychopathological symptoms (anxiety or depression) and those who are generally skeptical of vaccination.

## Introduction

Multiple sclerosis (MS) is the most common immune-mediated disease of the central nervous system that can lead to permanent disability^1^. There are worldwide around 2.8 million people affected by this disease. In Germany, approximately 280,000 people suffer from MS^2,3^. The etiology of MS has not been comprehensively clarified yet. In addition to genetic predisposition, the risk of MS is influenced by environmental and lifestyle factors such as vitamin D deficiency, nicotine abuse, obesity, and infection with Epstein-Barr virus^4–6^. MS is characterized by heterogeneous symptoms^7,8^.

Immunosuppressive as well as immunomodulatory disease-modifying therapies (DMTs) are widely used to treat MS. However, the use of certain DMTs, particularly B-cell-depleting therapies, is associated with an increased risk of infection. The highly contagious RNA virus severe acute respiratory syndrome type 2 (SARS‐CoV-2) can cause the coronavirus 2019 (COVID-19) disease. Severe courses of this disease can go along with a severe acute respiratory distress syndrome, which is associated with increased mortality and thus requires intensive medical treatment^9^. Therefore, the SARS-CoV-2 pandemic starting in 2020 and the risk of infection with SARS-CoV-2 posed a challenge to patients with MS^10–12^.

COVID-19 can include a variety of symptoms such as fever, sore throat, cough, chest and muscle pain, dyspnea, confusion, anosmia, ageusia, and headache^13^. The disease can progress to a life-threatening respiratory insufficiency also affecting the heart, kidney, liver, or the nervous system^14,15^. Treatment with DMTs in MS is associated with an increased risk of severe SARS-CoV-2 infection^16,17^. Studies have shown that an important risk factor for COVID-19 infection is the use of B-cell-depleting drugs^18^. It was shown that the frequency of having a severe clinical course of COVID-19 infection was 2–3 times higher with anti-CD20 therapy than with other DMTs^19^. A French study also showed that older age, male sex, obesity and a higher degree of disability are independent risk factors for SARS-CoV-2 infection^20^. More specifically, patients with an Expanded Disability Status Scale (EDSS) score higher than 6 had a sevenfold higher risk of suffering from a serious course of COVID-19^20^. The study also indicated that there is a 60% higher risk of having a severe course of COVID-19 for every 10 years of increasing age^20^.

The newly developed vaccines against SARS-CoV-2 played a central role in the global strategy to control the pandemic^21,22^. In Germany, four vaccines against SARS-CoV-2 had been approved by the European Medicines Agency (EMA) and recommended by the German Standing Committee on Vaccination (STIKO) of the Robert Koch Institute (RKI) at the time of this study: the mRNA-based vaccines tozinameran (BNT162b2, Comirnaty®) and elasomeran (mRNA-1273, Spikevax®), which contain specific mRNA molecules that encode protein antigens (Zhang et al. 2019), and the two vector-based vaccines AZD1222 (Vaxzevria®) and Ad26.COV2.S (Jcovden®)^23^.

Various sociodemographic and clinical factors as well as psychologic characteristics can impact the willingness or unwillingness to become vaccinated against SARS-CoV-2^24^. Risk perception and fear of infectious diseases also appear to be important predictors of vaccination acceptance^25^. A major reason for people to become vaccinated was to protect themselves and others from a severe COVID-19 course, but there were also concerns regarding the safety of the vaccines^26^. The occurrence of severe thrombotic events due to vaccination with AZD1222 in a number of cases led to further uncertainty in the population^27^. Although studies showed that SARS-CoV-2 vaccines are safe in patients with MS, with no increased risk of worsening of the disease or relapse^28,29^, the rapid development of vaccines and the lack of information and experience about (long-term) side effects of the vaccines administered were reasons leading to skepticism among patients with MS^30^, but also in the general population^31^. On the one hand, some patients with autoimmune diseases, such as MS, have initially raised concerns that disease progression may be exacerbated by vaccination^32^. On the other hand, MS patients have an increased risk of morbidity and mortality due to infections^33,34^. Even though numerous studies have disproved vaccinations as a trigger for MS or increased disease activity^35,36^, some MS patients have lasting concerns about being vaccinated^28^.

The aim of the present study was, on the one hand, to investigate whether a high general willingness to be vaccinated prior to the pandemic was associated with subsequent actual SARS-CoV-2 vaccination in patients with MS. On the other hand, we determined the reasons for or against SARS-CoV-2 vaccination with the new nucleic acid-based vaccines and how the patients' attitudes towards recommended standard vaccinations have developed during the pandemic. We also investigated the associations between psychological, sociodemographic, and clinical characteristics and the number of SARS-CoV-2 vaccinations received as well as the vaccination-related adverse events perceived.

## Methods

### Patient enrollment and consent

The data had been collected at two centers (Department of Neurology at the Rostock University Medical Center and Ecumenical Hainich Hospital Mühlhausen, Germany). Inclusion criteria for participants were an age ≥ 18 years and a confirmed diagnosis of a clinically isolated syndrome (CIS) or MS according to the revised McDonald criteria^37^. Written informed consent to participate in this study was obtained from all patients. Exclusion criteria were unwillingness to participate and diagnoses other than CIS or MS. The study was approved by the ethics committees of the University of Rostock (permit number A 2019-0048) and the State Medical Association of Thuringia and conducted in accordance with the Declaration of Helsinki and the European data protection regulations.

### Data collection

At baseline, sociodemographic data, e.g., age, sex, and educational level, as well as clinical data, e.g., course of MS, duration of disease, degree of disability according to the EDSS^38^, and comorbidities of the patients, have been acquired^24^. Comorbidities were categorized by organ system. These were as follows: cardiovascular, chronic inflammatory, pulmonary, neurological, metabolic, psychiatric, orthopedic, gastrointestinal, dermatological, ophthalmologic, otolaryngologic, urologic or gynecologic diseases, endocrinological, hematological, pain, cancer, and other diseases. The general willingness to receive the recommended vaccinations was surveyed in the form of a questionnaire since June 2019 and thus before the SARS-CoV-2 outbreak. The standard vaccinations (basic immunization and booster vaccinations) are listed in the STIKO vaccination calendar^39^. The Hospital Anxiety and Depression Scale (HADS) was used to assess psychological variables. The HADS is a self-report questionnaire to assess the severity of depression and anxiety. It consists of 14 questions that are answered on a 4-point Likert scale^40^. The classification of the patients was done according to Marrie et al.^41^: Scores of 0–7 points were considered normal, scores of 8–10 points were considered borderline, and scores of 11–21 were considered abnormal^42^. The data were collected during an ambulatory neurology appointment (outpatients) or during a hospital stay (inpatients). The baseline data were collected in Mühlhausen by JB and BS and in Rostock by PM, SEL and NF. Further details on the baseline data collection can be found in the published articles by Heidler et al.^43^ and Streckenbach et al.^44^. Of the original 404 patients in the baseline study, 211 patients did not participate in this follow-up study.

Approximately one year after the first COVID-19 vaccines were authorized in the EU (i.e., between October 2021 and the end of January 2022), we examined the patients about their SARS-CoV-2 vaccination status and vaccination-related adverse events. For the follow-up survey, the patients were asked the same set of questions in the same order in the form of a structured interview. The questionnaire consisted of 30 questions with sub-items. In some cases, more than one answer was possible. The data were collected and analyzed by PM, SEL and NF at the Department of Neurology at the Rostock University Medical Center. At the Ecumenical Hainich Hospital Mühlhausen, this was done by KB, JR and FH. We obtained information on administered vaccinations with the newly developed SARS-CoV-2 vaccines in the patients with MS. All vaccines that were approved in Germany up to the end of the survey period were recorded in the standardized interview: The two mRNA-based vaccines tozinameran and elasomeran and the two viral vector-based vaccines Ad26.COV2.S and AZD122. The protein-based vaccine NVX-CoV2373 (Nuvaxovid®), which was not available until the end of the survey period^45^, was not included in this study. The vaccinations were differentiated into first vaccination, second vaccination, and third vaccination. We also asked the changes of patients’ attitudes towards vaccination recommendations during the pandemic. Data on the perceived side effects after each SARS-CoV-2 vaccination administered were collected. A distinction was made between local (swelling, redness, or pain at the injection site) and systemic side effects (chest pain, fatigue, fever and chills, palpitations, headache, shortness of breath, muscle and joint pain, dizziness, malaise, or other adverse effects). The period for recording side effects after vaccination was not limited. In addition, the patients were asked about reasons for or against SARS-CoV-2 vaccination (multiple answers were possible) and whether they had experienced MS relapses or MS progression after a SARS-CoV-2 vaccination. In this context, self-reporting was used to ask after which vaccination a disease worsening or relapse occurred without specifying a time frame. It should be noted that due to the study design, the patients were not interviewed after a predetermined period of time following vaccination.

### Statistical analysis

The numerical and categorical data were recorded in SPSS. All data were pseudonymized. The statistical analyses were conducted using SPSS (version 27) and R (version 4.1.2). Descriptive statistics have been calculated for the sociodemographic and clinical data as well as for the vaccination data, including self-reported side effects. The chi-squared-test was used to test whether certain side effects occurred more or less frequently after administration of a particular SARS-CoV-2 vaccine as compared to other SARS-CoV-2 vaccines. Fisher’s exact test was used to evaluate the association between sex and the occurrence of side effects after SARS-CoV-2 vaccination. Binary logistic regression analyses were performed to assess the relationship of sociodemographic, clinical, and psychological variables as well as the pre-pandemic willingness to receive recommended standard vaccinations with SARS-CoV-2 vaccination status as well as the occurrence of side effects after SARS-CoV-2 vaccination. This yielded odds ratios (ORs) with 95% confidence intervals (CIs). As the number of patients with CIS was small, CIS and relapsing–remitting MS (RRMS) patients were combined for the statistical analysis. A* p*-value of < 0.05 was set as the criterion for statistical significance.

## Results

### Vaccination willingness and psychological characteristics of the patients

A total of 193 patients were included in this study (Table 1). The cohort was composed of patients with CIS (*n* = 12), RRMS (*n* = 121), secondary progressive MS (SPMS) (*n* = 48), and primary progressive MS (PPMS) (*n* = 12). The proportion of female patients was 67.9% (*n* = 131). A subset of 150 patients received a DMT for MS (77.7%). The DMTs and comorbidities of the patients are provided in Supplemental Tables S3 and S4.

**Table 1:** Characteristics of the patients (*N* = 193).

Characteristic	
Sex, *n* (%)	
Men	62 (32.1)
Women	131 (67.9)
Age (years), mean ± SD	48.0 ± 12.2
Education (years), mean ± SD	10.3 ± 1.1
Willingness to receive recommended vaccinations (pre-pandemic, *n* (%))	
Yes	146 (75.6)
No	47 (24.4)
Psychological variables^1^	
HADS-A score, *n* (%)	
Normal	98 (52.4)
Borderline	50 (26.7)
Abnormal	39 (20.9)
HADS-D score, *n* (%)	
Normal	128 (68.8)
Borderline	32 (17.2)
Abnormal	26 (14.0)
Disease duration (years), median (range)	9 (0–39)
Disease course, *n* (%)	
CIS/RRMS	133 (68.9)
SPMS	48 (24.9)
PPMS	12 (6.2)
EDSS score, mean ± SD	3.5 ± 2.3
Medical care, *n* (%)	
Inpatient	29 (15.0)
Outpatient	164 (85.0)
Use of DMT, *n* (%)	
Yes	150 (77.7)
No	43 (22.3)
Comorbidities, *n* (%)	
Yes	142 (73.6)
No	51 (26.4)
Prior infection with SARS-CoV-2^2^, *n* (%)	
Yes	10 (5.2)
No	183 (94.8)

*CIS* clinically isolated syndrome, *DMT* disease-modifying therapy approved for the treatment of multiple sclerosis, *EDSS* expanded disability status scale, *HADS-A* subscale of anxiety of the hospital anxiety and depression scale, *HADS-D* subscale of depression of the hospital anxiety and depression scale, *MS* multiple sclerosis, *n* number of patients, *PPMS* primary progressive MS, *RRMS* relapsing–remitting MS, *SARS-CoV-2* severe acute respiratory syndrome coronavirus 2, *SD* standard deviation, *SPMS* secondary progressive MS.

^1^There were missing values for HADS-A (*n* = 6) and HADS-D (*n* = 7). Only valid data were considered for the analyses.

^2^Prior to the survey regarding the SARS-CoV-2 vaccination status.

146 of the patients (75.6%) stated that they intend to have a complete standard vaccination status, i.e., that they are willing to follow the official vaccination recommendations in Germany^39^.

On the HADS-A scale, the score was in the normal range for 98 patients, in the borderline range for 50 patients, and in the abnormal range for 39 patients. A normal score was determined on the HADS-D scale for 128 patients. A borderline HADS-D score was obtained for 32 patients and an abnormal score for 26 patients. Seventeen out of the 26 (65.4%) patients with an abnormal HADS-D score also had an abnormal HADS-A score (Table 1).

### Reasons for or against SARS-CoV-2 vaccination

**Figure 1:**
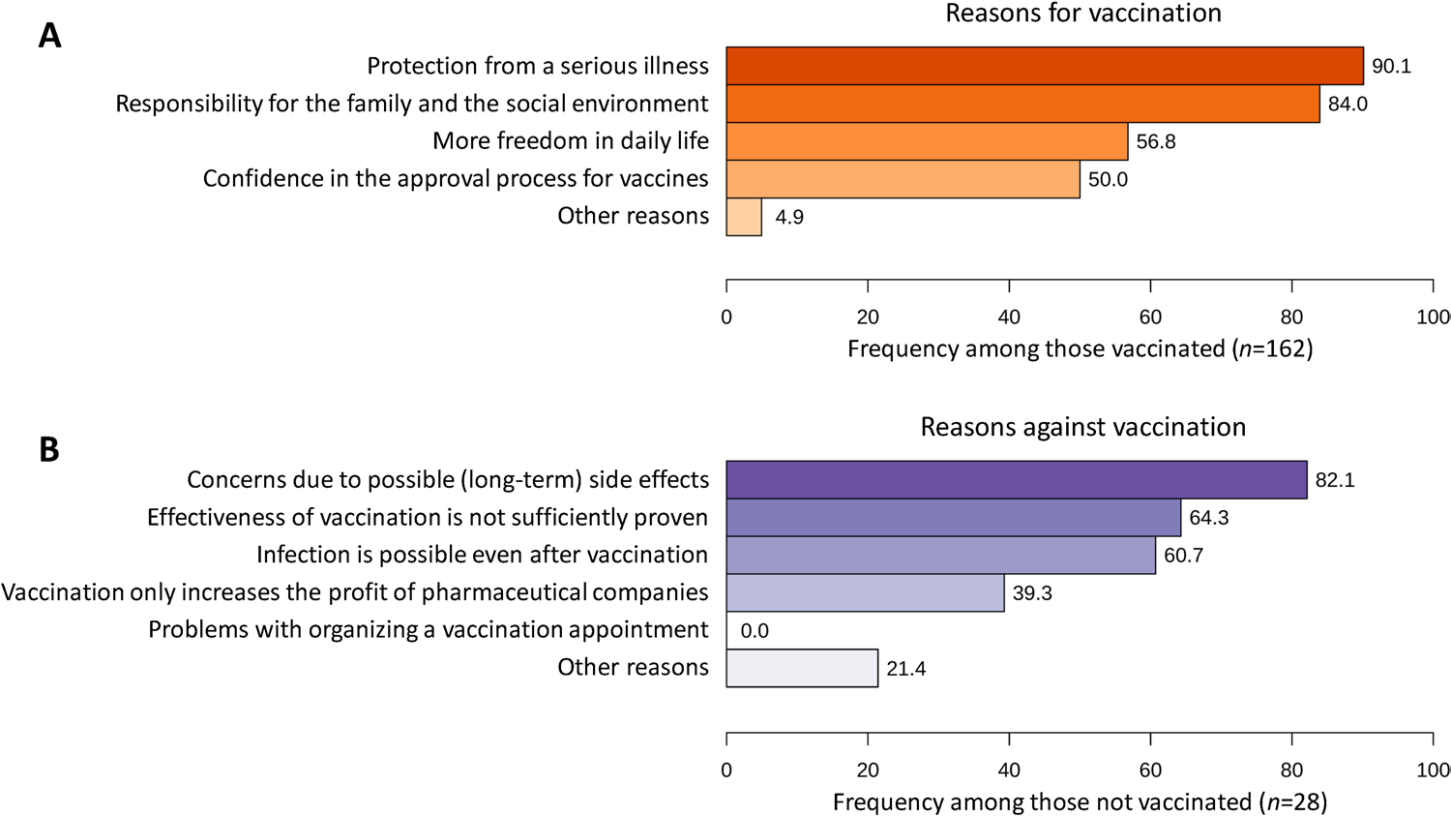
Prevalence of reasons for and against SARS-CoV-2 vaccination. MS patients with and without immunization against SARS-CoV-2 were asked about the reasons for (**A**) and against (**B**) the vaccination, respectively. It was possible to provide more than one reason. Three patients did not provide any information, which resulted in missing values. *MS* multiple sclerosis, *n* number of patients, *SARS-CoV-2* severe acute respiratory syndrome coronavirus 2.

Among the patients who received at least one vaccination against SARS-CoV-2, protection against a serious disease was the most common reason (90.1%). The most common reason against SARS-CoV-2 vaccination among the unvaccinated patients was concern about possible side effects (82.1%) (Fig. 1).

Of the 30 patients who were not vaccinated against SARS-CoV-2, eight reported that they had developed or increased a negative attitude toward standard vaccinations because of the coronavirus pandemic. Of the 30 unvaccinated patients, 16 patients (53.3%) had a positive attitude towards recommended standard vaccinations pre-pandemic. Of the 163 patients who were vaccinated at least once against SARS-CoV-2, 130 patients (79.8%) had a positive attitude toward the recommended standard vaccinations. In contrast, a negative attitude toward the recommended standard vaccinations prior to the coronavirus pandemic was reported by only 33 (20.2%) of the 163 patients who had received at least one vaccination against SARS-CoV-2 (Supplemental Table S2).

### SARS-CoV-2 vaccination status

Administration of the first, second, and third SARS-CoV-2 vaccine doses was reported by 163 (84.5%), 151 (78.2%) and 25 (13.0%) of the 193 MS patients, respectively (Table 2). There were 138 patients who had received only mRNA-based vaccines, and 13 patients had received only vector-based SARS-CoV-2 vaccines. At the second vaccine administration, 10 patients switched from previously receiving a vector-based vaccine to an mRNA-based vaccine. At the third vaccine administration, 2 patients switched to an mRNA vaccine and thus had a heterologous SARS-CoV-2 vaccination scheme. No patient switched from mRNA-based vaccines to vector-based vaccines. The date of the first SARS-CoV-2 vaccination was between January 2021 and January 2022.

**Table 2:** SARS-CoV-2 vaccination status of the patients with multiple sclerosis (*N* = 193).

SARS-CoV-2 vaccination^1^	1st vaccination	2nd vaccination	3rd vaccination^2^
Vaccination received, *n* (%)			
Yes	163 (84.5)	151 (78.2)	25 (13.0)
No	30 (15.5)	42 (21,8)	167 (87.0)
Type of vaccine received, *n* (%)			
Tozinameran	126 (77.3)	128 (84.8)	21 (84.0)
Elasomeran	12 (7.4)	13 (8.6)	4 (16.0)
AZD1222	18 (11.0)	9 (6.0)	0 (0)
Ad26.COV2.S	7 (4.3)	1 (0.7)	0 (0)

*N* number, *SARS-CoV-2* severe acute respiratory syndrome coronavirus 2.

^1^Vaccinations until the time point of data collection, which ended January 2022 (none of the patients had received more than three COVID-19 vaccinations).

^2^One case with missing data was not considered in the frequency tabulations.

### Factors associated with SARS-CoV-2 vaccine administration in patients with MS

Patients with a higher willingness to undergo the recommended standard vaccinations prior to the pandemic were significantly more likely to have received the first and second vaccination against SARS-CoV-2 (1st: OR = 3.447,* p* = 0.003; 2nd: OR = 3.155, *p* = 0.002) (Table 3). Conversely, patients with borderline HADS-D scores were significantly less likely to have been vaccinated against SARS-CoV-2. For the borderline and abnormal HADS-A and HADS-D categories, ORs < 1 consistently resulted for the first and second vaccinations, implicating that anxiety and depression were negatively associated with getting vaccinated against SARS-CoV-2 in general. There were no significant associations with sociodemographic data for any vaccination stage. Likewise, the type of MS course and the degree of disability (according to the EDSS) were not significantly associated with SARS-CoV-2 vaccinations. There was also no significant difference between the sexes with regard to the different vaccines administered (first SARS-CoV-2 vaccination: Fisher`s exact test *p* = 0.176, second SARS-CoV-2 vaccination: *p* = 0.171, third SARS-CoV-2 vaccination: *p* = 0.959). Disease duration was the only clinical variable that was significantly associated with receiving the third SARS-CoV-2 vaccination. More specifically, patients with longer disease duration were more likely to have received a third SARS-CoV-2 vaccination (OR = 1.051, *p* = 0.031) (Table 3). The comparison between SARS-CoV-2 vaccinated and unvaccinated patients in terms of individual characteristics can be found in Supplemental Table S2. Regarding DMTs and comorbidities, there was no significant difference between patients who had been vaccinated at least once against SARS-CoV-2 and unvaccinated patients (Supplemental Tables S3 and S4).

**Table 3:** Associations of SARS-CoV-2 vaccination status with patient characteristics and pre-pandemic standard vaccination willingness in patients with MS.

Parameter	1st vaccination			2nd vaccination			3rd vaccination		
	OR	95% conf. interv.	*p*-value	OR	95% conf. interv.	*p*-value	OR	95% conf. interv.	*p*-value
Sociodemographic data									
Sex (ref. male)	0.734	0.307–1.756	0.487	1.229	0.599–2.523	0.574	1.598	0.604–4.225	0.345
Age (in years)	1.027	0.994–1.060	0.108	1.018	0.989–1.046	0.223	0.999	0.966–1.034	0.973
Education (in years)	1.071	0.752–1.525	0.703	1.020	0.753–1.383	0.897	1.121	0.785–1.600	0.531
Willingness to receive recom-mended vaccinations	3.447	1.529–7.769	**0.003**	3.155	1.516–6.568	**0.002**	0.810	0.316–2.079	0.661
Psychological variables									
HADS-A (ref. normal)									
Borderline	0.542	0.223–1.318	0.177	0.659	0.300–1.451	0.301	1.066	0.370–3.074	0.906
Abnormal	0.841	0.295–2.398	0.746	0.994	0.396–2.492	0.989	1.150	0.372–3.556	0.809
HADS-D (ref. normal)									
Borderline	0.391	0.155–0.986	**0.047**	0.463	0.198–1.085	0.076	0.431	0.094–1.972	0.278
Abnormal	0.842	0.258–2.745	0.776	0.809	0.294–2.225	0.681	0.539	0.117–2.491	0.429
Clinical data									
Disease duration (in years)	0.984	0.942–1.027	0.455	1.004	0.965–1.045	0.847	1.051	1.004–1.099	**0.031**
Disease course (ref. CIS/RRMS)									
SPMS	1.098	0.435–2.776	0.843	1.262	0.550–2.898	0.583	1.280	0.491–3.335	0.614
PPMS	0.938	0.192–4.589	0.937	0.874	0.222–3.433	0.847	1.462	0.294–7.284	0.643
EDSS score	0.945	0.796–1.122	0.517	0.988	0.849–1.149	0.875	1.062	0.882–1.279	0.524
Medical care (ref. inpatient)	1.522	0.561–4.126	0.409	1.173	0.463–2.969	0.737	0.924	0.292–2.921	0.893
Use of DMT	1.626	0.683–3.871	0.272	1.548	0.712–3.368	0.270	1.178	0.415–3.349	0.758
Comorbidity (ref. no)	0.656	0.251–1.709	0.388	0.486	0.201–1.177	0.110	1.133	0.425–3.019	0.803
Prior infection with SARS-CoV-2 (ref. no infection)	1.695	0.207–13.892	0.623	0.393	0.106–1.464	0.163	0.731	0.089–6.034	0.771

*CIS* clinically isolated syndrome, *conf. interv.* confidence interval, *DMT* disease-modifying therapy approved for the treatment of multiple sclerosis, *EDSS* expanded disability status scale, *HADS-A* subscale of anxiety of the hospital anxiety and depression scale, *HADS-D* subscale of depression of the Hospital Anxiety and Depression Scale, *MS* multiple sclerosis, *OR* odds ratio from univariable logistic regression analysis, *PPMS* primary progressive MS, *ref.* reference, *RRMS* relapsing–remitting MS, *SARS-CoV-2* severe acute respiratory syndrome coronavirus 2, *SPMS* secondary progressive MS.

Significant values are given in bold.

### Side effects of SARS-CoV-2 vaccinations

**Figure 2:**
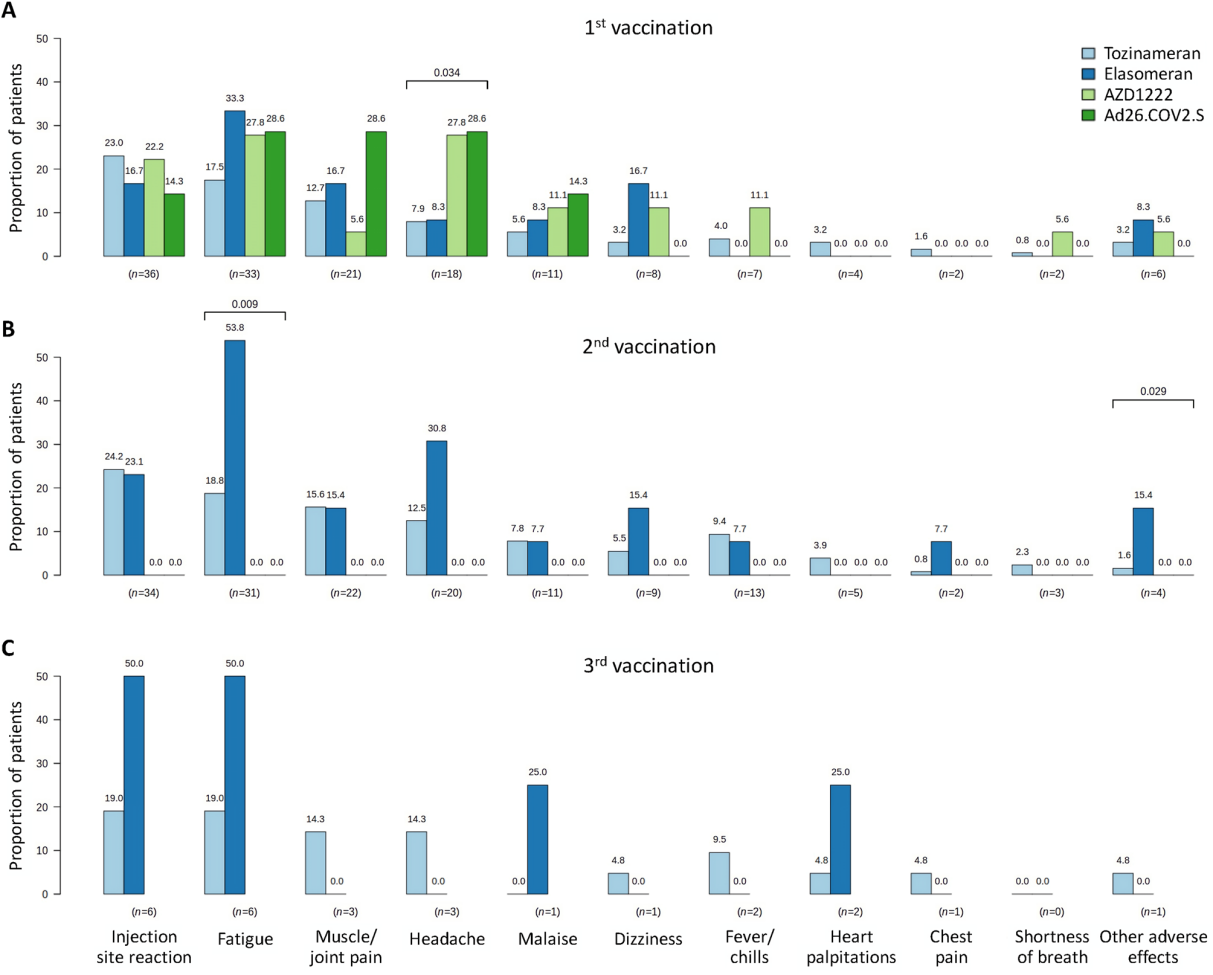
Adverse effects after SARS-CoV-2 vaccination in the patient cohort (*N* = 193). This figure shows the frequency of adverse effects (in descending order) after (**A**) the first (*n* = 163), (**B**) the second (*n* = 151) and (**C**) the third vaccination (*n* = 25). Significant differences between vaccine groups are indicated by brackets with chi-square test p-values (not adjusted for multiple testing) provided above. After the first SARS-CoV-2 vaccination, headache was more often reported by MS patients who received a vector-based vaccine (green bars), whereas fatigue was more often reported by patients who received elasomeran for the second vaccination (dark blue bar). However, it should be noted that the number of cases was sometimes small, in particular with regard to the third vaccination. *MS* multiple sclerosis, *n* number of patients, *SARS-CoV-2* severe acute respiratory syndrome coronavirus 2.

Of the 163 patients who received at least one SARS-CoV-2 vaccination, 52.8% reported an adverse reaction. A subset of 44.2% of the patients reported systemic side effects, and 28.8% of the patients reported local side effects after any vaccination. After administration of the first SARS-CoV-2 vaccination, 39.9% of the patients reported side effects. The most frequently reported systemic side effects after the first vaccination, regardless of the vaccine type administered, were fatigue (20.2%), muscle and joint pain (12.9%), and headache (11.0%). After the second vaccination with elasomeran, fever or chills and chest pain occurred in some patients, unlike after the first administration of elasomeran. Moreover, the rate of fatigue after the second elasomeran administration was 53.8% and thus considerately higher than after the first vaccination with elasomeran. Regarding AZD1222, the most frequently reported adverse events after the first vaccination were headache and fatigue, each affecting 27.8% of the patients. In comparison, for Ad26.COV2.S, the most common side effects were fatigue, headache, and muscle/joint pain. Common side effects of tozinameran were fatigue and injection site reactions. There were only minor differences in the side effect profiles when comparing the four vaccines analyzed. Patients who initially received a vector-based vaccine reported headache significantly more frequently than patients who initially received an mRNA-based vaccine (chi-squared test *p* = 0.034). On the other hand, after the second vaccination against SARS-CoV-2, complaints about fatigue were reported only by patients who received mRNA vaccines (*p* = 0.009) (Fig. 2).

### Relapse or worsening of MS after SARS-CoV-2 vaccination

Only few patients (*n* = 7, *n* = 5, *n* = 0 after the first, second, and third vaccination, respectively) self-reported that they had experienced relapse activity or disease worsening after a SARS-CoV-2 vaccination. Among them, six patients reported worsening after the first administration of tozinameran and one patient after the first administration of AZD1222. The five patients who reported disease worsening after the second SARS-CoV-2 vaccination received tozinameran. Of the patients who reported a relapse or worsening of their disease, eight were taking DMTs.

### Factors associated with side effects following SARS-CoV-2 vaccine administration in patients with MS

Overall, 61.5% of the women and 35.2% of the men reported side effects after any SARS-CoV-2 vaccination (Fisher’s exact test *p* = 0.003). Women were more likely than men to report headache (*p* = 0.008) and muscle/joint pain (*p* = 0.023) after the second vaccination than men.

Further analyses of psychological, sociodemographic, and clinical data in relation to specific adverse events following individual administrations of SARS-CoV-2 vaccines showed nominally statistically significant associations for HADS-A score and HADS-D score, age, education, EDSS score, disease course, disease duration, DMT utilization, and previous SARS-CoV-2 infection (Supplemental Table S1). For example, it was found that patients with abnormal HADS-D scores reported significantly more often that they experienced dizziness after the initial SARS-CoV-2 vaccination (OR = 5.944, *p* = 0.018). Patients who were not willing to receive recommended standard vaccinations at baseline also reported significantly more often dizziness after the first SARS-CoV-2 vaccination (OR = 4.345, *p* = 0.046).

There was an association between younger age and perception of fatigue symptoms after the first SARS-CoV-2 vaccination (OR: 0.969, 95% CI 0.939–1.000, *p* = 0.049). Following the second SARS-CoV-2 vaccination, fever or chills (OR: 0.923, 95% CI 0.876–0.973, *p* = 0.003), headache (OR: 0.954, 95% CI 0.917–0.992, *p* = 0.020), and muscle and joint pain (OR: 0.954, 95% CI 0.918–0.991, *p* = 0.015) occurred more frequently in patients of younger age. MS disease duration was positively associated with self-reported heart palpitations after the first SARS-CoV-2 immunization (OR: 1.110, 95% CI 1.004–1.226, *p* = 0.041), while it was negatively associated with fatigue (OR: 0.944, 95% CI 0.894–0.997, *p* = 0.038), headache (OR: 0.917, 95% CI 0.852–0.988, *p* = 0.023), fever/chills (OR: 0.893, 95% CI 0.806–0.989, *p* = 0.031) as well as muscle and joint pain (OR: 0.915, 95% CI 0.852–0.984, *p* = 0.016) after the second SARS-CoV-2 vaccination. PPMS patients did not show a significant difference in the occurrence of post-vaccination adverse events. Patients with SPMS, however, were less likely to experience muscle and joint pain (OR: 0.116, 95% CI 0.015–0.901, *p* = 0.039) and fatigue (OR: 0.260, 95% CI 0.074–0.918, *p* = 0.036) after the second SARS-CoV-2 vaccination than patients with CIS or RRMS (Supplemental Table S1).

Only one of the 163 patients vaccinated against SARS-CoV-2 reported developing an anti-attitude towards standard vaccinations in the course of the pandemic. This patient experienced fatigue, headache, and malaise following both the first and second SARS-CoV-2 vaccination.

## Discussion

Given the increased risk of infection in MS patients treated with DMTs^46–49^, the enhancement of immune response against pathogens is an important issue^28,50,51^. In this study, we provide information on the actual SARS-CoV-2 vaccination rate in MS patients approximately one year after the approval of first vaccines and the vaccination side effect profile of the patients in relation to the pre-pandemic willingness to receive recommended standard vaccinations as well as psychological, sociodemographic, and clinical variables.

More than three quarters (84.5%) of the surveyed patients received at least one SARS-CoV-2 vaccination. This is similar to the actual vaccination rate of the general population in Germany (78.0%) reported by the RKI^52^. There was also no apparent difference in the rate of receiving a second vaccination in our study as compared to the general population (78.2% vs. 76.4%). Regarding the frequency of the third vaccination, no adequate comparison with the general population could be made due to the survey period, as most patients (87.0%) had not yet received a third SARS-CoV-2 vaccination at the time of the survey, presumably because a booster vaccination was not yet recommended for most of them as the second vaccination was less than 6 months ago^53^.

This study revealed a relatively high actual SARS-CoV-2 vaccination rate compared to 65% of MS patients with willingness to become vaccinated against SARS-CoV-2 in a previous study by Heidler et al.^24^. We were able to show that patients who were willing to receive the recommended standard vaccinations prior to the pandemic were also more often vaccinated against SARS-CoV-2. The positive vaccination attitude seems to be a predictor for the acceptance of the novel SARS-CoV-2 vaccines. Other reasons for this could be extensive education and information campaigns as well as low-threshold offers to become vaccinated. However, political incentives during the SARS-CoV-2 pandemic, such as fewer restrictions in everyday life for vaccinated people, may also have had a positive influence on the decision to become vaccinated. In addition, a prior positive SARS-CoV-2 infection test positively influenced the vaccination setting^54^. We were able to show that a key reason for SARS-CoV-2 vaccination was protection against severe disease. A study by Rzymski et al. revealed that 88% of immunosuppressed patients, representing a group at high risk of severe COVID-19 courses, were willing to receive a booster vaccination against SARS-CoV-2^55^. Furthermore, studies have shown that older age leads to higher vaccination propensity towards SARS-CoV-2 vaccines^56–59^. This has already been shown in other studies for influenza vaccination^60^. It would be reasonable to postulate that patients of older age could be more attentive to their healthcare and that physicians pay more attention to the complete vaccination status of this patient group. A survey in Germany also showed that men and older people were more likely to be vaccinated against SARS-CoV-2^54^. According to RKI data, 90.1% of people over 60 years of age in Germany received a basic immunization against SARS-CoV-2^61^. This could be due to the vaccination recommendation of the STIKO with its phased prioritization plan in Germany: People with old age as well as patients with a particular high risk to suffer from a serious COVID-19 course preferentially received a vaccine against SARS-CoV-2 in the initial phase^62^. Another reason that older patients were more likely to be vaccinated may be that older age is associated with a higher likelihood of comorbidities (*r* = 0.32, *p* < 0.001), which are known to be a risk factor for more severe outcomes^63^ and therefore may be linked to a higher vaccination acceptance.

Our data suggest that sex is not an important factor affecting SARS-CoV-2 vaccination coverage. However, studies from other countries such as Australia and Israel indicated that women are more reluctant to receive SARS-CoV-2 vaccines than men^64,65^. The way people are informed about vaccinations might influence the willingness to receive a vaccine and thus also the actual vaccination rate. High exposure to conflicting information from social media as well as low trust in the healthcare system are factors associated with a negative attitude towards acceptance of SARS-CoV-2 vaccination. In addition, an overabundance of information and news can trigger a type of fatigue and desensitization^66,67^. Conversely, a high level of trust in governmental institutions as well as in scientifically based sources of information and better trust in the healthcare system promotes a higher propensity to become vaccinated^68^. This could be a starting point for improving vaccination rates by making information transparently available to the population in a simple and understandable way^58^. Positively influencing factors on vaccination also include higher education levels^69^. Other related factors include how patients perceive their exposure and the exposure of others to COVID-19. Patients with a chronic disease as well as patients who perceived increased risk potential to contract COVID-19 in their personal environment reported a higher willingness to receive SARS-CoV-2 vaccination^70–72^. We found that patients with borderline HADS-D scores were significantly less likely to be vaccinated against SARS-CoV-2. This could be due to the fact that patients who are more depressed have less motivation and drive to get vaccinated. A negative association between vaccination acceptance and depression was also found in other studies^73,74^. One possible explanation could be that patients with depressive symptoms are more likely to believe misinformation about SARS-CoV-2 vaccinations^75^. In general, patients with mental illness were more likely to have an incomplete vaccination status^76^. Policies like having more freedom in daily life after full immunization are associated with increased vaccination willingness^77^. In our study population, this was also a major reason for getting vaccinated against SARS-CoV-2 (56.8%). The main reason for patients who did not get vaccinated against SARS-CoV-2 was the unforeseeable risk of short-term or long-term side effects (82.1%). This corresponds to other studies, according to which patients with vaccination hesitancy frequently expressed concerns about the efficacy and safety of the SARS-CoV-2 vaccines^78^. Another factor is concern about quality control, rapid development, and the side effect profile of SARS-CoV-2 vaccines^79^. In a survey representative of the general population, the main reasons for hesitating to vaccinate were that vaccines have not been adequately tested, fear of side effects, and a desire to act at one's own discretion^80^.

In our study, we also examined the usage frequency of the different SARS-CoV-2 vaccines available. Tozinameran was administered most frequently for basic immunization and booster vaccination. In contrast, the AZD1222 vaccine showed a decline in vaccination coverage from first to third vaccination. For the third vaccination, this agent was no longer used in the patients in our study. The reason for this was presumably a STIKO recommendation and the fact that, due to its side effect profile, the vaccine has only been recommended for certain patient groups in Germany since December 1, 2021. Unusual thrombotic events have been reported in association with the use of AZD1222^81–83^.

Furthermore, we analyzed the side effect profile of the SARS-CoV-2 vaccines. A proportion of 28.8% of the patients reported local general reactions after vaccine administration, which included swelling, pain, and redness at the injection site. This result is similar to the study by Archiron et al. in which 555 patients with MS were interviewed regarding their side effects after the first and second SARS-CoV-2 vaccination: 16% reported local pain at the injection site after the first vaccination and 14.2% after the second vaccination^29^. Among systemic reactions reported by our study population, fatigue was the most common symptom. This is also in line with the above-mentioned study by Archiron et al.: 9.2% and 15.9% of MS patients reported fatigue as a side effect after the first and second dose of tozinameran, respectively^29^. In the pivotal efficacy trial, which included 21,720 participants who received tozinameran, fatigue and headache were the most common systemic vaccination reactions (59% and 52%, respectively)^84^. In a recent study involving MS patients from the United Kingdom and Germany, the most common side effects after SARS-CoV-2 vaccination were headache, fatigue, and pain at the injection site^85^. Women were more frequently affected by these side effects than men, which is in line with our study. Regarding the overall occurrence of side effects in our cohort, there were associations with age, sex, EDSS score and the course of the disease. Patients with abnormal HADS scores more often reported muscle and joint pain and dizziness after SARS-CoV-2 vaccination. Patients who were already skeptical of recommended standard vaccinations before the pandemic also reported dizziness after SARS-CoV-2 vaccination more frequently. This raises the question of whether these patients pay more attention to side effects after getting vaccinated against SARS-CoV-2. Furthermore, our results showed a significant association between self-reported palpitations after the first vaccination and years of education. This may suggest that the patients with higher levels of education were more attentive to or better able to remember side effects such as palpitations, which have been previously reported for mRNA vaccines^86,87^. Another consideration for the results is that patients with SPMS tend to be older than patients with CIS or RRMS. It could therefore be hypothesized that the natural aging of the immune system leads to a decrease in the immune response, and therefore patients with SPMS may report less often side effects (fatigue and joint and muscle pain) than patients with CIS or RRMS^88^. However, some of the results were close to *p* = 0.05, which may indicate statistical associations but not necessarily clinically meaningful associations. On the other hand, the associations mostly reached statistical significance after the second SARS-CoV-2 vaccination, but not after the first or third vaccination. These and other inconsistencies in our analysis of associations between patient characteristics and the occurrence of adverse effects after individual SARS-CoV-2 vaccinations may be indicative of false positive findings, and therefore our results should be interpreted with caution. Nevertheless, our data suggest that patients with MS are not at an increased risk of experiencing side effects following SARS-CoV-2 vaccination as compared to the general population.

After any SARS-CoV-2 vaccination, less than 5% of our patients reported an exacerbation of the disease. These data are similar to another study of MS patients from Germany according to which the frequency of reported relapses after SARS-CoV-2 vaccination was 9.3%^89^. In a study by Achiron et al., the rate of patients with an acute relapse was 2.1%^29^. In another study, 1661 MS patients were evaluated after SARS-CoV-2 vaccination^90^. That study showed a slight increase in the annualized relapse rate after the vaccination as compared to the previous year (0.22 vs. 0.17)^90^. In another analysis of 425 MS patients, 136 patients reported adverse events after SARS-CoV-2 vaccination and 36 patients reported new or worsening neurological symptoms^91^. However, most symptoms disappeared after 24 h to 3 days. A recent study showed that mRNA-based SARS-CoV-2 vaccination in MS patients did not result in worsening of the autoimmune disease nor triggered immune-mediated neurological diseases^92^.

The lack of a control group from the general population could be interpreted as a limitation of the present study. Furthermore, although we conducted a standardized interview, the validity of our data might be limited by factors such as recall bias and the inherent subjectivity of self-reports. It should be also noted that the patients were interviewed after a variable period of time after the last vaccination (if they had received any). However, the study examined important clinical aspects that were not addressed in the pivotal studies of the SARS-CoV-2 vaccines in patients with autoimmune disease, especially for patients with MS. In addition, the patient cohort was examined regarding their willingness to follow standard vaccination recommendations and the attitude change during the course of the pandemic.

## Conclusion

This study has shown that MS patients were relatively likely to get vaccinated against SARS-CoV-2 despite their concerns about getting vaccinated. We showed that patients who had a positive vaccination attitude prior to the pandemic were 3 times more likely to become vaccinated against SARS-CoV-2 in the course of the pandemic. In our studies, the leading reason for vaccination was protection against severe disease. In contrast, the leading reason against SARS-CoV-2 vaccination was the concern about possible side effects. With regard to the psychological characteristics, patients with borderline HADS-D score were less likely to be vaccinated against SARS-CoV-2. We did not find associations between sociodemographic and clinical variables and the likelihood of getting vaccinated against SARS-CoV-2, except for MS disease duration. Approximately half of the patients reported side effects after SARS-CoV-2 vaccination, independently of the type of vaccine administered. The leading side effects were fatigue, muscle and joint pain, headache, and redness at the injection site. Women reported side effects significantly more frequently than men. Younger patient age and shorter disease duration were also associated with an increased likelihood of certain side effects. These data suggest that it would be important to pay special attention to patients who exhibit vaccine skepticism to increase their vaccine acceptance through targeted counseling. An interesting research topic for further investigation is how SARS-CoV-2 vaccination rates evolved in the context of recommended booster vaccinations and after protein-based vaccines became available, particularly in older people and patients with chronic disease such as MS.

### Supplementary Information

Supplementary Table 1.

Supplementary Table 2.

Supplementary Table 3.

Supplementary Table 4.

## Data Availability

The datasets generated during and/or analysed during the current study are available from the corresponding author on reasonable request.
