# Supplementary material for: Vaccination status and self-reported side effects after SARS-CoV-2 vaccination in relation to psychological and clinical variables in patients with multiple sclerosis
Source: Sci Rep. 2024 May 28;14:12248. doi: 10.1038/s41598-024-62541-x (PMC11133397; doi:10.1038/s41598-024-62541-x)
Supplement: Supplementary file 2 — Supplementary Table 2. [file 41598_2024_62541_MOESM2_ESM.docx]

**Supplemental Table S2: Comparison between SARS-CoV-2 vaccinated and unvaccinated patients in terms of patient characteristics and** **pre-pandemic readiness for standard vaccinations in patients with MS**

| **Parameter** | **Vaccinated** | **Unvaccinated** | ***p*-value** |
| --- | --- | --- | --- |
| **Sex, *n* (%)**  Men  Women | 54 (33.1)  109 (66.9) | 8 (26.7)  22 (73.3) | 0.532^3^ |
| **Age (years), mean ± SD** | 48.6 ± 12.3 | 44.7 ± 11.3 | 0.091^4^ |
| **Education (years), mean ± SD** | 10.3 ± 1.2 | 10.2 ± 0.7 | 0.584^4^ |
| **Willingness to receive recommended vaccinations (pre-pandemic, *n* (%))**  Yes  No | 130 (79.8)  33 (20.2) | 16 (53.3)  14 (46.7) | **0.004**^3^ |
| **Psychological variables^1^**  HADS-A score, *n* (%)  Normal  Borderline  Abnormal  HADS-D score, *n* (%)  Normal  Borderline  Abnormal | 85 (54.1)  39 (24.8)  33 (21.0)  111 (71.2)  23 (14.7)  22 (14.1) | 13 (43.3)  11 (36.7)  6 (20.0)  17 (56.7)  9 (30.0)  4 (13.3) | 0.388^5^  0.124^5^ |
| **Disease duration (years), median (range)** | 9 (0-39) | 9.5 (1-37) | 0.278^6^ |
| **Disease course, *n* (%)**  CIS/RRMS  SPMS  PPMS | 112 (68.7%)  41 (25.2%)  10 (6.1%) | 21 (70.0%)  7 (23.3%)  2 (6.7%) | 0.975^5^ |
| **EDSS score, mean ± SD** | 3.4 ± 2.3 | 3.7 ± 2.4 | 0.535^4^ |
| **Medical care, *n* (%)**  Inpatient  Outpatient | 23 (14.1%)  140 (85.9%) | 6 (20.0%)  24 (80.0%) | 0.409^3^ |
| **Use of DMT, *n* (%)**  Yes  No | 129 (79.1)  34 (20.9) | 21 (70.0)  9 (30.0) | 0.338^3^ |
| **Comorbidities, *n* (%)**  Yes  No | 118 (72.4%)  45 (27.6%) | 24 (80.0%)  6 (20.0%) | 0.501^3^ |
| **Prior infection with SARS-CoV-2^2^, *n* (%)**  Yes  No | 9 (5.5%)  154 (94.5%) | 1 (3.3%)  29 (96.7%) | 1.000^3^ |

CIS = clinically isolated syndrome; DMT = disease-modifying therapy approved for the treatment of multiple sclerosis; EDSS = Expanded Disability Status Scale; HADS-A = subscale of anxiety of the Hospital Anxiety and Depression Scale, HADS-D = subscale of depression of the Hospital Anxiety and Depression Scale; MS = multiple sclerosis; *n* = number of patients; PPMS = primary progressive MS; RRMS = relapsing-remitting MS; SARS-CoV-2 = severe acute respiratory syndrome coronavirus 2; SD = standard deviation; SPMS = secondary progressive MS.

^1^ There were missing values for HADS-A (*n* = 6) and HADS-D (*n* = 7). Only valid data were considered for the analyses.

^2^ Prior to the survey regarding the SARS-CoV-2 vaccination status**.**

^3^ Fisher's exact test

^4^ Welch's t-test

^5^ Chi square-test

^6^ Mann-Whitney U test
