# Supplementary material for: Vaccination status and self-reported side effects after SARS-CoV-2 vaccination in relation to psychological and clinical variables in patients with multiple sclerosis
Source: Sci Rep. 2024 May 28;14:12248. doi: 10.1038/s41598-024-62541-x (PMC11133397; doi:10.1038/s41598-024-62541-x)
Supplement: Supplementary file 3 — Supplementary Table 3. [file 41598_2024_62541_MOESM3_ESM.docx]

**Supplemental Table S3: Distribution of DMT use for the total cohort and in comparison of MS patients with and without vaccination against SARS-CoV-2**

| **DMTs** | **Total**  ***N* (%)** | **Vaccinated *N* (%)** | **Unvaccinated *N* (%)** | ***p*-value^1^** |
| --- | --- | --- | --- | --- |
| Interferon beta | 28 (14.5) | 26 (16.0) | 2 (6.7) | 0.262 |
| Glatiramer acetate | 21 (10.9) | 20 (12.3) | 1 (3.3) | 0.208 |
| Ocrelizumab | 18 (9.3) | 16 (9.8) | 2 (6.7) | 0.744 |
| Teriflunomide | 16 (8.3) | 13 (8.0) | 3 (10.0) | 0.719 |
| Natalizumab | 16 (8.3) | 12 (7.4) | 4 (13.3) | 0.282 |
| Pulsed corticosteroids | 16 (8.3) | 12 (7.4) | 4 (13.3) | 0.282 |
| Fingolimod | 15 (7.8) | 13 (8.0) | 2 (6.7) | 1.000 |
| Dimethyl fumarate | 11 (5.7) | 9 (5.5) | 2 (6.7) | 0.682 |
| Mitoxantrone | 3 (1.6) | 3 (1.8) | 0 (0.0) | 1.000 |
| Cladribine | 3 (1.6) | 2 (1.2) | 1 (3.3) | 0.399 |
| Alemtuzumab | 1 (0.5) | 1 (0.6) | 0 (0.0) | 1.000 |
| Intravenous immunoglobulin G | 1 (0.5) | 1 (0.6) | 0 (0.0) | 1.000 |
| Azathioprine | 1 (0.5) | 1 (0.6) | 0 (0.0) | 1.000 |

^1^ Fisher's exact test
