# Supplementary material for: Vaccination status and self-reported side effects after SARS-CoV-2 vaccination in relation to psychological and clinical variables in patients with multiple sclerosis
Source: Sci Rep. 2024 May 28;14:12248. doi: 10.1038/s41598-024-62541-x (PMC11133397; doi:10.1038/s41598-024-62541-x)
Supplement: Supplementary file 4 — Supplementary Table 4. [file 41598_2024_62541_MOESM4_ESM.docx]

**Supplemental Table S4: Frequency of comorbidities for the total cohort and in comparison of MS patients with and without vaccination against SARS-CoV-2**

| **Comorbidities** | **Total**  ***N* (%)** | **Vaccinated**  ***N* (%)** | **Unvaccinated**  ***N* (%)** | ***p*-value^1^** |
| --- | --- | --- | --- | --- |
| Cardiovascular diseases | 65 (33.7) | 56 (34.4) | 9 (30.0) | 0.834 |
| Other inflammatory diseases | 5 (2.6) | 5 (3.1) | 0 (0.0) | 1.000 |
| Pulmonary diseases | 8 (4.1) | 6 (3.7) | 2 (6.7) | 0.361 |
| Neurological diseases | 22 (11.4) | 17 (10.4) | 5 (16.7) | 0.348 |
| Metabolic diseases | 43 (22.3) | 36 (22.1) | 7 (23.3) | 0.816 |
| Psychiatric diseases | 39 (20.2) | 34 (20.9) | 5 (16.7) | 0.805 |
| Orthopedic diseases | 29 (15.0) | 27 (16.5) | 2 (6.7) | 0.264 |
| Gastrointestinal diseases | 27 (14.0) | 22 (13.5) | 5 (16.7) | 0.579 |
| Dermatologic diseases | 12 (6.2) | 9 (5.5) | 3 (10.0) | 0.404 |
| Ophthalmologic diseases | 7 (3.6) | 7 (4.3) | 0 (0.0) | 0.598 |
| Otolaryngologic diseases | 1 (0.5) | 1 (0.6) | 0 (0.0) | 1.000 |
| Urologic or gynecologic diseases | 22 (11.4) | 15 (9.2) | 7 (23.3) | 0.053 |
| Endocrinological diseases | 21 (10.9) | 15 (9.2) | 6 (20.0) | 0.106 |
| Pain | 4 (2.1) | 2 (1.2) | 2 (6.7) | 0.115 |
| Cancer | 2 (1.0) | 2 (1.2) | 0 (0.0) | 1.000 |
| Hematological diseases | 4 (2.1) | 2 (1.2) | 2 (6.7) | 0.115 |
| Other diseases | 17 (8.8) | 13 (8.0) | 4 (13.3) | 0.309 |

^1^ Fisher's exact test
